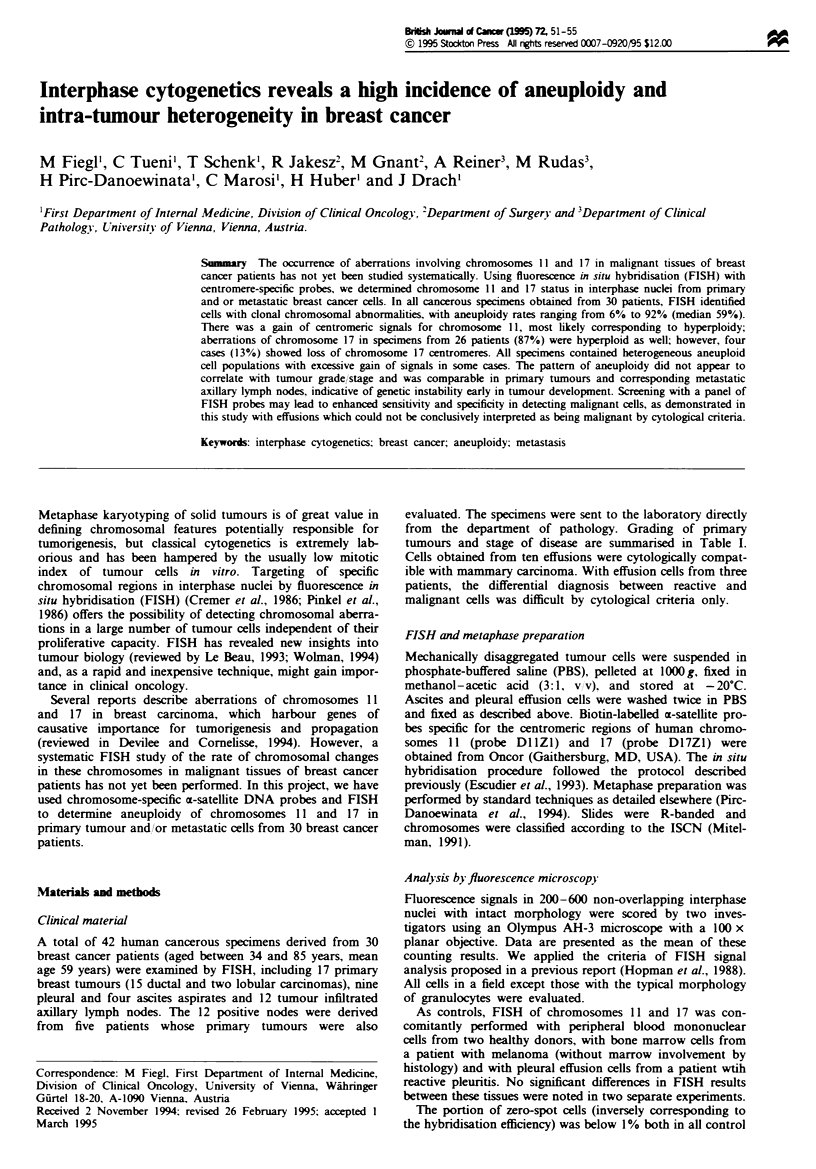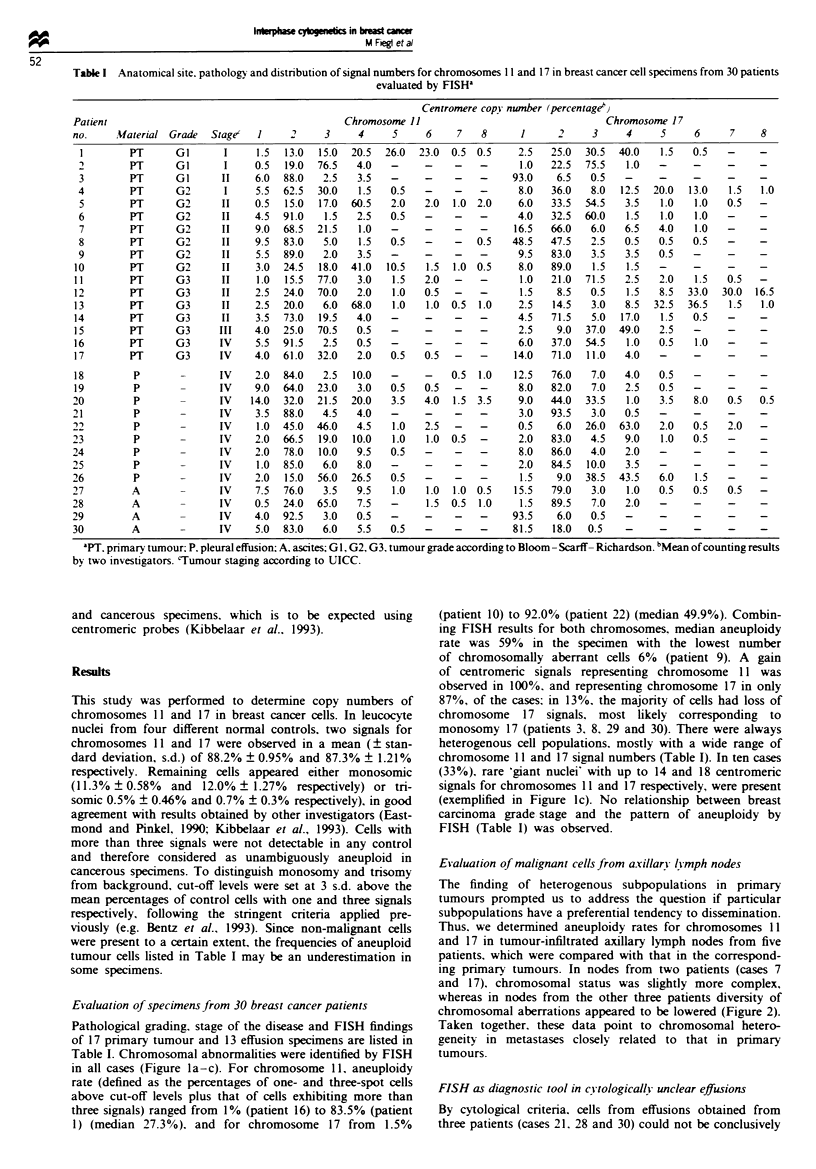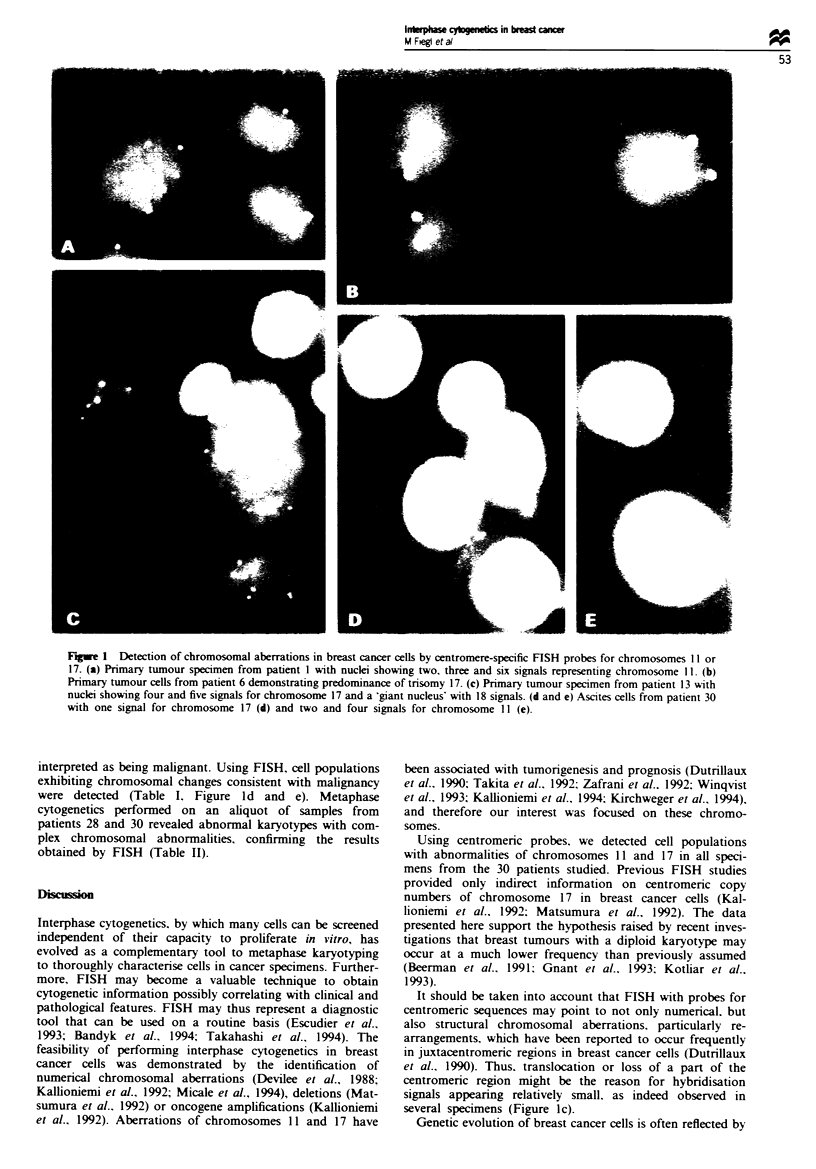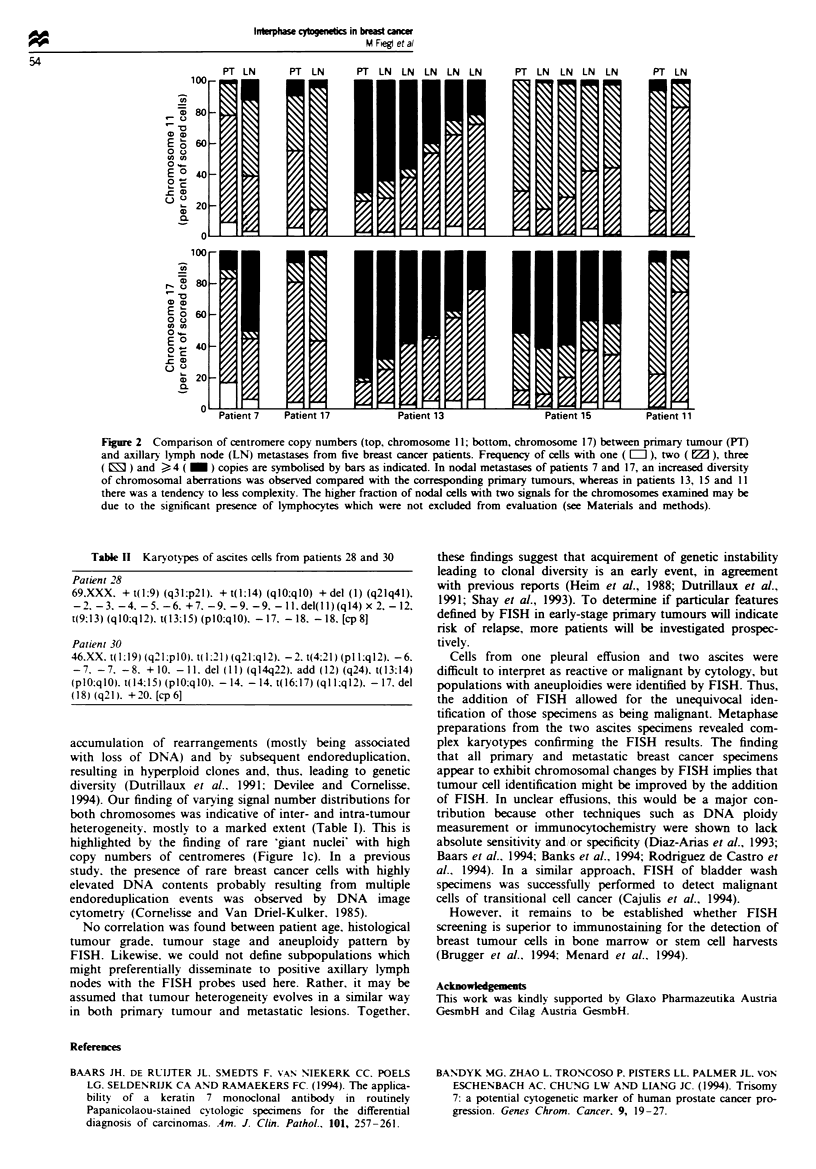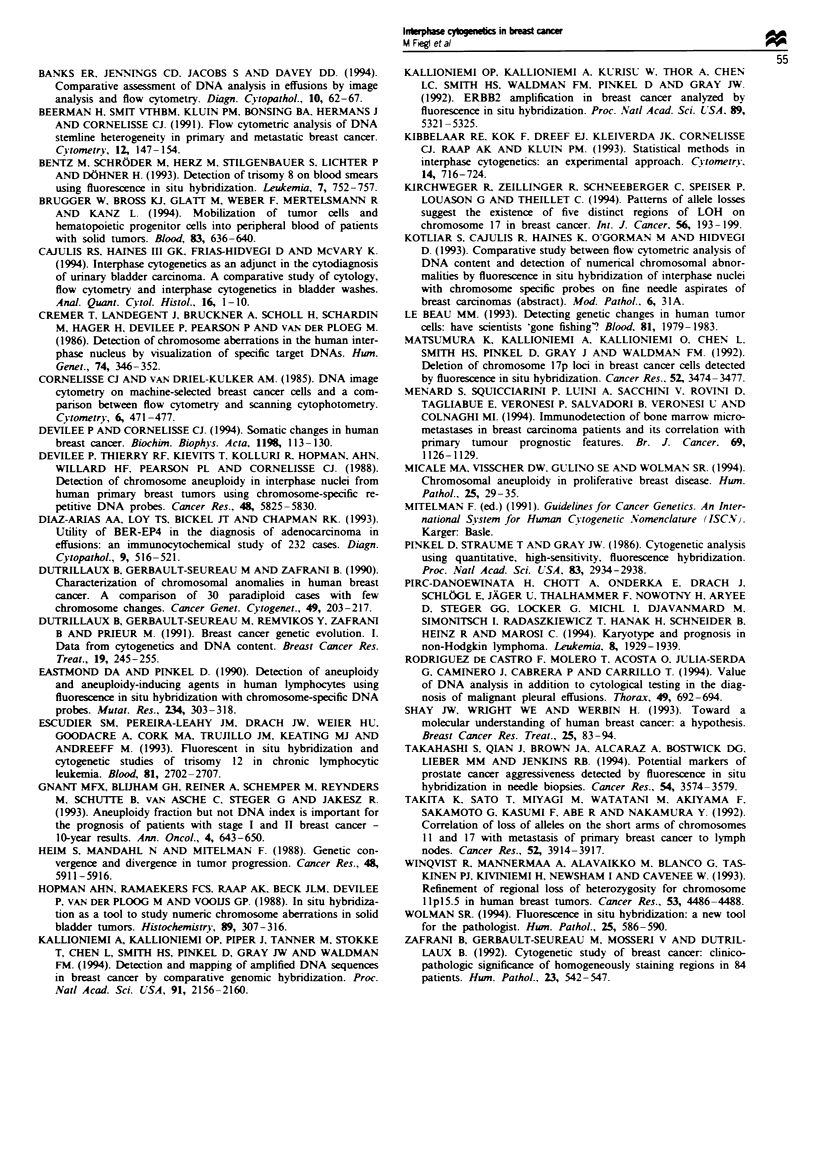# Interphase cytogenetics reveals a high incidence of aneuploidy and intra-tumour heterogeneity in breast cancer.

**DOI:** 10.1038/bjc.1995.276

**Published:** 1995-07

**Authors:** M. Fiegl, C. Tueni, T. Schenk, R. Jakesz, M. Gnant, A. Reiner, M. Rudas, H. Pirc-Danoewinata, C. Marosi, H. Huber

**Affiliations:** First Department of Internal Medicine, University of Vienna, Austria.

## Abstract

**Images:**


British Journal of Cancer (1995) 72, 51-55

© 1995 Stockton Press All rights reserved 0007-0920/95 $12.00

### Interphase cytogenetics reveals a high incidence of aneuploidy and intra-tumour heterogeneity in breast cancer

M Fiegl[1], C Tueni[1], T Schenk[1], R Jakesz[2], M Gnant[2], A Reiner[3], M Rudas[3], H Pirc-Danoewinata[1], C Marosi[1], H Huber[1] and J Drach[1]

[1]*First Department of Internal Medicine, Division of Clinical Oncology,* [2]*Department of Surgery and* [3]*Department of Clinical Pathology, University of Vienna, Vienna, Austria.*

**Summary** The occurrence of aberrations involving chromosomes 11 and 17 in malignant tissues of breast cancer patients has not yet been studied systematically. Using fluorescence *in situ* hybridisation (FISH) with centromere-specific probes, we determined chromosome 11 and 17 status in interphase nuclei from primary and/or metastatic breast cancer cells. In all cancerous specimens obtained from 30 patients, FISH identified cells with clonal chromosomal abnormalities, with aneuploidy rates ranging from 6% to 92% (median 59%). There was a gain of centromeric signals for chromosome 11, most likely corresponding to hyperploidy; aberrations of chromosome 17 in specimens from 26 patients (87%) were hyperploid as well; however, four cases (13%) showed loss of chromosome 17 centromeres. All specimens contained heterogeneous aneuploid cell populations with excessive gain of signals in some cases. The pattern of aneuploidy did not appear to correlate with tumour grade/stage and was comparable in primary tumours and corresponding metastatic axillary lymph nodes, indicative of genetic instability early in tumour development. Screening with a panel of FISH probes may lead to enhanced sensitivity and specificity in detecting malignant cells, as demonstrated in this study with effusions which could not be conclusively interpreted as being malignant by cytological criteria.

**Keywords**: interphase cytogenetics; breast cancer; aneuploidy; metastasis

Metaphase karyotyping of solid tumours is of great value in defining chromosomal features potentially responsible for tumorigenesis, but classical cytogenetics is extremely laborious and has been hampered by the usually low mitotic index of tumour cells *in vitro*. Targeting of specific chromosomal regions in interphase nuclei by fluorescence *in situ* hybridisation (FISH) (Cremer *et al.*, 1986; Pinkel *et al.*, 1986) offers the possibility of detecting chromosomal aberrations in a large number of tumour cells independent of their proliferative capacity. FISH has revealed new insights into tumour biology (reviewed by Le Beau, 1993; Wolman, 1994) and, as a rapid and inexpensive technique, might gain importance in clinical oncology.

Several reports describe aberrations of chromosomes 11 and 17 in breast carcinoma, which harbour genes of causative importance for tumorigenesis and propagation (reviewed in Devilee and Cornelisse, 1994). However, a systematic FISH study of the rate of chromosomal changes in these chromosomes in malignant tissues of breast cancer patients has not yet been performed. In this project, we have used chromosome-specific α-satellite DNA probes and FISH to determine aneuploidy of chromosomes 11 and 17 in primary tumour and/or metastatic cells from 30 breast cancer patients.

### Materials and methods

### Clinical material

A total of 42 human cancerous specimens derived from 30 breast cancer patients (aged between 34 and 85 years, mean age 59 years) were examined by FISH, including 17 primary breast tumours (15 ductal and two lobular carcinomas), nine pleural and four ascites aspirates and 12 tumour infiltrated axillary lymph nodes. The 12 positive nodes were derived from five patients whose primary tumours were also evaluated. The specimens were sent to the laboratory directly from the department of pathology. Grading of primary tumours and stage of disease are summarised in Table I. Cells obtained from ten effusions were cytologically compatible with mammary carcinoma. With effusion cells from three patients, the differential diagnosis between reactive and malignant cells was difficult by cytological criteria only.

### FISH and metaphase preparation

Mechanically disaggregated tumour cells were suspended in phosphate-buffered saline (PBS), pelleted at 1000 *g*, fixed in methanol-acetic acid (3:1, v/v), and stored at −20°C. Ascites and pleural effusion cells were washed twice in PBS and fixed as described above. Biotin-labelled α-satellite probes specific for the centromeric regions of human chromosomes 11 (probe D11Z1) and 17 (probe D17Z1) were obtained from Oncor (Gaithersburg, MD, USA). The *in situ* hybridisation procedure followed the protocol described previously (Escudier *et al.*, 1993). Metaphase preparation was performed by standard techniques as detailed elsewhere (Pirc-Danoewinata *et al.*, 1994). Slides were R-banded and chromosomes were classified according to the ISCN (Mitelman, 1991).

### Analysis by fluorescence microscopy

Fluorescence signals in 200-600 non-overlapping interphase nuclei with intact morphology were scored by two investigators using an Olympus AH-3 microscope with a 100 × planar objective. Data are presented as the mean of these counting results. We applied the criteria of FISH signal analysis proposed in a previous report (Hopman *et al.*, 1988). All cells in a field except those with the typical morphology of granulocytes were evaluated.

As controls, FISH of chromosomes 11 and 17 was concomitantly performed with peripheral blood mononuclear cells from two healthy donors, with bone marrow cells from a patient with melanoma (without marrow involvement by histology) and with pleural effusion cells from a patient wtih reactive pleuritis. No significant differences in FISH results between these tissues were noted in two separate experiments.

The portion of zero-spot cells (inversely corresponding to the hybridisation efficiency) was below 1% both in all control

Correspondence: M Fiegl, First Department of Internal Medicine, Division of Clinical Oncology, University of Vienna, Währinger Gürtel 18-20, A-1090 Vienna, Austria
Received 2 November 1994; revised 26 February 1995; accepted 1 March 1995

**Table I** Anatomical site, pathology and distribution of signal numbers for chromosomes 11 and 17 in breast cancer cell specimens from 30 patients evaluated by FISH[a]

| Patient no. | Material | Grade | Stage[c] | Centromere copy number (percentage[b]) Chromosome 11: 1 | 2 | 3 | 4 | 5 | 6 | 7 | 8 | Chromosome 17: 1 | 2 | 3 | 4 | 5 | 6 | 7 | 8 |
|---|---|---|---|---|---|---|---|---|---|---|---|---|---|---|---|---|---|---|---|
| 1 | PT | G1 | I | 1.5 | 13.0 | 15.0 | 20.5 | 26.0 | 23.0 | 0.5 | 0.5 | 2.5 | 25.0 | 30.5 | 40.0 | 1.5 | 0.5 | – | – |
| 2 | PT | G1 | I | 0.5 | 19.0 | 76.5 | 4.0 | – | – | – | – | 1.0 | 22.5 | 75.5 | 1.0 | – | – | – | – |
| 3 | PT | G1 | II | 6.0 | 88.0 | 2.5 | 3.5 | – | – | – | – | 93.0 | 6.5 | 0.5 | – | – | – | – | – |
| 4 | PT | G2 | I | 5.5 | 62.5 | 30.0 | 1.5 | 0.5 | – | – | – | 8.0 | 36.0 | 8.0 | 12.5 | 20.0 | 13.0 | 1.5 | 1.0 |
| 5 | PT | G2 | II | 0.5 | 15.0 | 17.0 | 60.5 | 2.0 | 2.0 | 1.0 | 2.0 | 6.0 | 33.5 | 54.5 | 3.5 | 1.0 | 1.0 | 0.5 | – |
| 6 | PT | G2 | II | 4.5 | 91.0 | 1.5 | 2.5 | 0.5 | – | – | – | 4.0 | 32.5 | 60.0 | 1.5 | 1.0 | 1.0 | – | – |
| 7 | PT | G2 | II | 9.0 | 68.5 | 21.5 | 1.0 | – | – | – | – | 16.5 | 66.0 | 6.0 | 6.5 | 4.0 | 1.0 | – | – |
| 8 | PT | G2 | II | 9.5 | 83.0 | 5.0 | 1.5 | 0.5 | – | – | 0.5 | 48.5 | 47.5 | 2.5 | 0.5 | 0.5 | 0.5 | – | – |
| 9 | PT | G2 | II | 5.5 | 89.0 | 2.0 | 3.5 | – | – | – | – | 9.5 | 83.0 | 3.5 | 3.5 | 0.5 | – | – | – |
| 10 | PT | G2 | II | 3.0 | 24.5 | 18.0 | 41.0 | 10.5 | 1.5 | 1.0 | 0.5 | 8.0 | 89.0 | 1.5 | 1.5 | – | – | – | – |
| 11 | PT | G3 | II | 1.0 | 15.5 | 77.0 | 3.0 | 1.5 | 2.0 | – | – | 1.0 | 21.0 | 71.5 | 2.5 | 2.0 | 1.5 | 0.5 | – |
| 12 | PT | G3 | II | 2.5 | 24.0 | 70.0 | 2.0 | 1.0 | 0.5 | – | – | 1.5 | 8.5 | 0.5 | 1.5 | 8.5 | 33.0 | 30.0 | 16.5 |
| 13 | PT | G3 | II | 2.5 | 20.0 | 6.0 | 68.0 | 1.0 | 1.0 | 0.5 | 1.0 | 2.5 | 14.5 | 3.0 | 8.5 | 32.5 | 36.5 | 1.5 | 1.0 |
| 14 | PT | G3 | II | 3.5 | 73.0 | 19.5 | 4.0 | – | – | – | – | 4.5 | 71.5 | 5.0 | 17.0 | 1.5 | 0.5 | – | – |
| 15 | PT | G3 | III | 4.0 | 25.0 | 70.5 | 0.5 | – | – | – | – | 2.5 | 9.0 | 37.0 | 49.0 | 2.5 | – | – | – |
| 16 | PT | G3 | IV | 5.5 | 91.5 | 2.5 | 0.5 | – | – | – | – | 6.0 | 37.0 | 54.5 | 1.0 | 0.5 | 1.0 | – | – |
| 17 | PT | G3 | IV | 4.0 | 61.0 | 32.0 | 2.0 | 0.5 | 0.5 | – | – | 14.0 | 71.0 | 11.0 | 4.0 | – | – | – | – |
| 18 | P | – | IV | 2.0 | 84.0 | 2.5 | 10.0 | – | – | 0.5 | 1.0 | 12.5 | 76.0 | 7.0 | 4.0 | 0.5 | – | – | – |
| 19 | P | – | IV | 9.0 | 64.0 | 23.0 | 3.0 | 0.5 | 0.5 | – | – | 8.0 | 82.0 | 7.0 | 2.5 | 0.5 | – | – | – |
| 20 | P | – | IV | 14.0 | 32.0 | 21.5 | 20.0 | 3.5 | 4.0 | 1.5 | 3.5 | 9.0 | 44.0 | 33.5 | 1.0 | 3.5 | 8.0 | 0.5 | 0.5 |
| 21 | P | – | IV | 3.5 | 88.0 | 4.5 | 4.0 | – | – | – | – | 3.0 | 93.5 | 3.0 | 0.5 | – | – | – | – |
| 22 | P | – | IV | 1.0 | 45.0 | 46.0 | 4.5 | 1.0 | 2.5 | – | – | 0.5 | 6.0 | 26.0 | 63.0 | 2.0 | 0.5 | 2.0 | – |
| 23 | P | – | IV | 2.0 | 66.5 | 19.0 | 10.0 | 1.0 | 1.0 | 0.5 | – | 2.0 | 83.0 | 4.5 | 9.0 | 1.0 | 0.5 | – | – |
| 24 | P | – | IV | 2.0 | 78.0 | 10.0 | 9.5 | 0.5 | – | – | – | 8.0 | 86.0 | 4.0 | 2.0 | – | – | – | – |
| 25 | P | – | IV | 1.0 | 85.0 | 6.0 | 8.0 | – | – | – | – | 2.0 | 84.5 | 10.0 | 3.5 | – | – | – | – |
| 26 | P | – | IV | 2.0 | 15.0 | 56.0 | 26.5 | 0.5 | – | – | – | 1.5 | 9.0 | 38.5 | 43.5 | 6.0 | 1.5 | – | – |
| 27 | A | – | IV | 7.5 | 76.0 | 3.5 | 9.5 | 1.0 | 1.0 | 1.0 | 0.5 | 15.5 | 79.0 | 3.0 | 1.0 | 0.5 | 0.5 | 0.5 | – |
| 28 | A | – | IV | 0.5 | 24.0 | 65.0 | 7.5 | – | 1.5 | 0.5 | 1.0 | 1.5 | 89.5 | 7.0 | 2.0 | – | – | – | – |
| 29 | A | – | IV | 4.0 | 92.5 | 3.0 | 0.5 | – | – | – | – | 93.5 | 6.0 | 0.5 | – | – | – | – | – |
| 30 | A | – | IV | 5.0 | 83.0 | 6.0 | 5.5 | 0.5 | – | – | – | 81.5 | 18.0 | 0.5 | – | – | – | – | – |

[a]PT, primary tumour; P, pleural effusion; A, ascites; G1, G2, G3, tumour grade according to Bloom–Scarff–Richardson. [b]Mean of counting results by two investigators. [c]Tumour staging according to UICC.

and cancerous specimens, which is to be expected using centromeric probes (Kibbelaar *et al*., 1993).

### Results

This study was performed to determine copy numbers of chromosomes 11 and 17 in breast cancer cells. In leucocyte nuclei from four different normal controls, two signals for chromosomes 11 and 17 were observed in a mean (± standard deviation, s.d.) of 88.2% ± 0.95% and 87.3% ± 1.21% respectively. Remaining cells appeared either monosomic (11.3% ± 0.58% and 12.0% ± 1.27% respectively) or trisomic 0.5% ± 0.46% and 0.7% ± 0.3% respectively), in good agreement with results obtained by other investigators (Eastmond and Pinkel, 1990; Kibbelaar *et al*., 1993). Cells with more than three signals were not detectable in any control and therefore considered as unambiguously aneuploid in cancerous specimens. To distinguish monosomy and trisomy from background, cut-off levels were set at 3 s.d. above the mean percentages of control cells with one and three signals respectively, following the stringent criteria applied previously (e.g. Bentz *et al*., 1993). Since non-malignant cells were present to a certain extent, the frequencies of aneuploid tumour cells listed in Table I may be an underestimation in some specimens.

### *Evaluation of specimens from 30 breast cancer patients*

Pathological grading, stage of the disease and FISH findings of 17 primary tumour and 13 effusion specimens are listed in Table I. Chromosomal abnormalities were identified by FISH in all cases (Figure 1a–c). For chromosome 11, aneuploidy rate (defined as the percentages of one- and three-spot cells above cut-off levels plus that of cells exhibiting more than three signals) ranged from 1% (patient 16) to 83.5% (patient 1) (median 27.3%), and for chromosome 17 from 1.5% (patient 10) to 92.0% (patient 22) (median 49.9%). Combining FISH results for both chromosomes, median aneuploidy rate was 59% in the specimen with the lowest number of chromosomally aberrant cells 6% (patient 9). A gain of centromeric signals representing chromosome 11 was observed in 100%, and representing chromosome 17 in only 87%, of the cases; in 13%, the majority of cells had loss of chromosome 17 signals, most likely corresponding to monosomy 17 (patients 3, 8, 29 and 30). There were always heterogenous cell populations, mostly with a wide range of chromosome 11 and 17 signal numbers (Table I). In ten cases (33%), rare 'giant nuclei' with up to 14 and 18 centromeric signals for chromosomes 11 and 17 respectively, were present (exemplified in Figure 1c). No relationship between breast carcinoma grade stage and the pattern of aneuploidy by FISH (Table I) was observed.

### *Evaluation of malignant cells from axillary lymph nodes*

The finding of heterogenous subpopulations in primary tumours prompted us to address the question if particular subpopulations have a preferential tendency to dissemination. Thus, we determined aneuploidy rates for chromosomes 11 and 17 in tumour-infiltrated axillary lymph nodes from five patients, which were compared with that in the corresponding primary tumours. In nodes from two patients (cases 7 and 17), chromosomal status was slightly more complex, whereas in nodes from the other three patients diversity of chromosomal aberrations appeared to be lowered (Figure 2). Taken together, these data point to chromosomal heterogeneity in metastases closely related to that in primary tumours.

### *FISH as diagnostic tool in cytologically unclear effusions*

By cytological criteria, cells from effusions obtained from three patients (cases 21, 28 and 30) could not be conclusively

**Figure 1** Detection of chromosomal aberrations in breast cancer cells by centromere-specific FISH probes for chromosomes 11 or 17. (**a**) Primary tumour specimen from patient 1 with nuclei showing two, three and six signals representing chromosome 11. (**b**) Primary tumour cells from patient 6 demonstrating predominance of trisomy 17. (**c**) Primary tumour specimen from patient 13 with nuclei showing four and five signals for chromosome 17 and a 'giant nucleus' with 18 signals. (**d** and **e**) Ascites cells from patient 30 with one signal for chromosome 17 (**d**) and two and four signals for chromosome 11 (**e**).

interpreted as being malignant. Using FISH, cell populations exhibiting chromosomal changes consistent with malignancy were detected (Table I, Figure 1d and e). Metaphase cytogenetics performed on an aliquot of samples from patients 28 and 30 revealed abnormal karyotypes with complex chromosomal abnormalities, confirming the results obtained by FISH (Table II).

### Discussion

Interphase cytogenetics, by which many cells can be screened independent of their capacity to proliferate *in vitro*, has evolved as a complementary tool to metaphase karyotyping to thoroughly characterise cells in cancer specimens. Furthermore, FISH may become a valuable technique to obtain cytogenetic information possibly correlating with clinical and pathological features. FISH may thus represent a diagnostic tool that can be used on a routine basis (Escudier *et al.*, 1993; Bandyk *et al.*, 1994; Takahashi *et al.*, 1994). The feasibility of performing interphase cytogenetics in breast cancer cells was demonstrated by the identification of numerical chromosomal aberrations (Devilee *et al.*, 1988; Kallioniemi *et al.*, 1992; Micale *et al.*, 1994), deletions (Matsumura *et al.*, 1992) or oncogene amplifications (Kallioniemi *et al.*, 1992). Aberrations of chromosomes 11 and 17 have been associated with tumorigenesis and prognosis (Dutrillaux *et al.*, 1990; Takita *et al.*, 1992; Zafrani *et al.*, 1992; Winqvist *et al.*, 1993; Kallioniemi *et al.*, 1994; Kirchweger *et al.*, 1994), and therefore our interest was focused on these chromosomes.

Using centromeric probes, we detected cell populations with abnormalities of chromosomes 11 and 17 in all specimens from the 30 patients studied. Previous FISH studies provided only indirect information on centromeric copy numbers of chromosome 17 in breast cancer cells (Kallioniemi *et al.*, 1992; Matsumura *et al.*, 1992). The data presented here support the hypothesis raised by recent investigations that breast tumours with a diploid karyotype may occur at a much lower frequency than previously assumed (Beerman *et al.*, 1991; Gnant *et al.*, 1993; Kotliar *et al.*, 1993).

It should be taken into account that FISH with probes for centromeric sequences may point to not only numerical, but also structural chromosomal aberrations, particularly rearrangements, which have been reported to occur frequently in juxtacentromeric regions in breast cancer cells (Dutrillaux *et al.*, 1990). Thus, translocation or loss of a part of the centromeric region might be the reason for hybridisation signals appearing relatively small, as indeed observed in several specimens (Figure 1c).

Genetic evolution of breast cancer cells is often reflected by

**Figure 2** Comparison of centromere copy numbers (top, chromosome 11; bottom, chromosome 17) between primary tumour (PT) and axillary lymph node (LN) metastases from five breast cancer patients. Frequency of cells with one ( □ ), two ( ▨ ), three ( ▧ ) and ≥4 ( ■ ) copies are symbolised by bars as indicated. In nodal metastases of patients 7 and 17, an increased diversity of chromosomal aberrations was observed compared with the corresponding primary tumours, whereas in patients 13, 15 and 11 there was a tendency to less complexity. The higher fraction of nodal cells with two signals for the chromosomes examined may be due to the significant presence of lymphocytes which were not excluded from evaluation (see Materials and methods).

**Table II** Karyotypes of ascites cells from patients 28 and 30

*Patient 28*

69,XXX, + t(1;9) (q31;p21), + t(1;14) (q10;q10) + del (1) (q21q41), −2, −3, −4, −5, −6, +7, −9, −9, −9, −11, del(11)(q14) x 2, −12, t(9;13) (q10;q12), t(13;15) (p10;q10), −17, −18, −18, [cp 8]

*Patient 30*

46,XX, t(1;19) (q21;p10), t(1;21) (q21;q12), −2, t(4;21) (p11;q12), −6, −7, −7, −8, +10, −11, del (11) (q14q22), add (12) (q24), t(13;14) (p10;q10), t(14;15) (p10;q10), −14, −14, t(16;17) (q11;q12), −17, del (18) (q21), +20, [cp 6]

accumulation of rearrangements (mostly being associated with loss of DNA) and by subsequent endoreduplication, resulting in hyperploid clones and, thus, leading to genetic diversity (Dutrillaux *et al.*, 1991; Devilee and Cornelisse, 1994). Our finding of varying signal number distributions for both chromosomes was indicative of inter- and intra-tumour heterogeneity, mostly to a marked extent (Table I). This is highlighted by the finding of rare 'giant nuclei' with high copy numbers of centromeres (Figure 1c). In a previous study, the presence of rare breast cancer cells with highly elevated DNA contents probably resulting from multiple endoreduplication events was observed by DNA image cytometry (Cornelisse and Van Driel-Kulker, 1985).

No correlation was found between patient age, histological tumour grade, tumour stage and aneuploidy pattern by FISH. Likewise, we could not define subpopulations which might preferentially disseminate to positive axillary lymph nodes with the FISH probes used here. Rather, it may be assumed that tumour heterogeneity evolves in a similar way in both primary tumour and metastatic lesions. Together, these findings suggest that acquirement of genetic instability leading to clonal diversity is an early event, in agreement with previous reports (Heim *et al.*, 1988; Dutrillaux *et al.*, 1991; Shay *et al.*, 1993). To determine if particular features defined by FISH in early-stage primary tumours will indicate risk of relapse, more patients will be investigated prospectively.

Cells from one pleural effusion and two ascites were difficult to interpret as reactive or malignant by cytology, but populations with aneuploidies were identified by FISH. Thus, the addition of FISH allowed for the unequivocal identification of those specimens as being malignant. Metaphase preparations from the two ascites specimens revealed complex karyotypes confirming the FISH results. The finding that all primary and metastatic breast cancer specimens appear to exhibit chromosomal changes by FISH implies that tumour cell identification might be improved by the addition of FISH. In unclear effusions, this would be a major contribution because other techniques such as DNA ploidy measurement or immunocytochemistry were shown to lack absolute sensitivity and/or specificity (Diaz-Arias *et al.*, 1993; Baars *et al.*, 1994; Banks *et al.*, 1994; Rodriguez de Castro *et al.*, 1994). In a similar approach, FISH of bladder wash specimens was successfully performed to detect malignant cells of transitional cell cancer (Cajulis *et al.*, 1994).

However, it remains to be established whether FISH screening is superior to immunostaining for the detection of breast tumour cells in bone marrow or stem cell harvests (Brugger *et al.*, 1994; Menard *et al.*, 1994).

**Acknowledgements**
This work was kindly supported by Glaxo Pharmazeutika Austria GesmbH and Cilag Austria GesmbH.

**References**

BAARS JH, DE RUIJTER JL, SMEDTS F, VAN NIEKERK CC, POELS LG, SELDENRIJK CA AND RAMAEKERS FC. (1994). The applicability of a keratin 7 monoclonal antibody in routinely Papanicolaou-stained cytologic specimens for the differential diagnosis of carcinomas. *Am. J. Clin. Pathol.*, **101**, 257–261.

BANDYK MG, ZHAO L, TRONCOSO P, PISTERS LL, PALMER JL, VON ESCHENBACH AC, CHUNG LW AND LIANG JC. (1994). Trisomy 7: a potential cytogenetic marker of human prostate cancer progression. *Genes Chrom. Cancer*, **9**, 19–27.

BANKS ER, JENNINGS CD, JACOBS S AND DAVEY DD. (1994). Comparative assessment of DNA analysis in effusions by image analysis and flow cytometry. *Diagn. Cytopathol.*, **10**, 62-67.

BEERMAN H, SMIT VTHBM, KLUIN PM, BONSING BA, HERMANS J AND CORNELISSE CJ. (1991). Flow cytometric analysis of DNA stemline heterogeneity in primary and metastatic breast cancer. *Cytometry*, **12**, 147-154.

BENTZ M, SCHRÖDER M, HERZ M, STILGENBAUER S, LICHTER P AND DÖHNER H. (1993). Detection of trisomy 8 on blood smears using fluorescence in situ hybridization. *Leukemia*, **7**, 752-757.

BRUGGER W, BROSS KJ, GLATT M, WEBER F, MERTELSMANN R AND KANZ L. (1994). Mobilization of tumor cells and hematopoietic progenitor cells into peripheral blood of patients with solid tumors. *Blood*, **83**, 636-640.

CAJULIS RS, HAINES III GK, FRIAS-HIDVEGI D AND MCVARY K. (1994). Interphase cytogenetics as an adjunct in the cytodiagnosis of urinary bladder carcinoma. A comparative study of cytology, flow cytometry and interphase cytogenetics in bladder washes. *Anal. Quant. Cytol. Histol.*, **16**, 1-10.

CREMER T, LANDEGENT J, BRUCKNER A, SCHOLL H, SCHARDIN M, HAGER H, DEVILEE P, PEARSON P AND VAN DER PLOEG M. (1986). Detection of chromosome aberrations in the human interphase nucleus by visualization of specific target DNAs. *Hum. Genet.*, **74**, 346-352.

CORNELISSE CJ AND VAN DRIEL-KULKER AM. (1985). DNA image cytometry on machine-selected breast cancer cells and a comparison between flow cytometry and scanning cytophotometry. *Cytometry*, **6**, 471-477.

DEVILEE P AND CORNELISSE CJ. (1994). Somatic changes in human breast cancer. *Biochim. Biophys. Acta*, **1198**, 113-130.

DEVILEE P, THIERRY RF, KIEVITS T, KOLLURI R, HOPMAN, AHN, WILLARD HF, PEARSON PL AND CORNELISSE CJ. (1988). Detection of chromosome aneuploidy in interphase nuclei from human primary breast tumors using chromosome-specific repetitive DNA probes. *Cancer Res.*, **48**, 5825-5830.

DIAZ-ARIAS AA, LOY TS, BICKEL JT AND CHAPMAN RK. (1993). Utility of BER-EP4 in the diagnosis of adenocarcinoma in effusions: an immunocytochemical study of 232 cases. *Diagn. Cytopathol.*, **9**, 516-521.

DUTRILLAUX B, GERBAULT-SEUREAU M AND ZAFRANI B. (1990). Characterization of chromosomal anomalies in human breast cancer. A comparison of 30 paradiploid cases with few chromosome changes. *Cancer Genet. Cytogenet.*, **49**, 203-217.

DUTRILLAUX B, GERBAULT-SEUREAU M, REMVIKOS Y, ZAFRANI B AND PRIEUR M. (1991). Breast cancer genetic evolution. I. Data from cytogenetics and DNA content. *Breast Cancer Res. Treat.*, **19**, 245-255.

EASTMOND DA AND PINKEL D. (1990). Detection of aneuploidy and aneuploidy-inducing agents in human lymphocytes using fluorescence in situ hybridization with chromosome-specific DNA probes. *Mutat. Res.*, **234**, 303-318.

ESCUDIER SM, PEREIRA-LEAHY JM, DRACH JW, WEIER HU, GOODACRE A, CORK MA, TRUJILLO JM, KEATING MJ AND ANDREEFF M. (1993). Fluorescent in situ hybridization and cytogenetic studies of trisomy 12 in chronic lymphocytic leukemia. *Blood*, **81**, 2702-2707.

GNANT MFX, BLIJHAM GH, REINER A, SCHEMPER M, REYNDERS M, SCHUTTE B, VAN ASCHE C, STEGER G AND JAKESZ R. (1993). Aneuploidy fraction but not DNA index is important for the prognosis of patients with stage I and II breast cancer - 10-year results. *Ann. Oncol.*, **4**, 643-650.

HEIM S, MANDAHL N AND MITELMAN F. (1988). Genetic convergence and divergence in tumor progression. *Cancer Res.*, **48**, 5911-5916.

HOPMAN AHN, RAMAEKERS FCS, RAAP AK, BECK JLM, DEVILEE P, VAN DER PLOOG M AND VOOIJS GP. (1988). In situ hybridization as a tool to study numeric chromosome aberrations in solid bladder tumors. *Histochemistry*, **89**, 307-316.

KALLIONIEMI A, KALLIONIEMI OP, PIPER J, TANNER M, STOKKE T, CHEN L, SMITH HS, PINKEL D, GRAY JW AND WALDMAN FM. (1994). Detection and mapping of amplified DNA sequences in breast cancer by comparative genomic hybridization. *Proc. Natl Acad. Sci. USA*, **91**, 2156-2160.

KALLIONIEMI OP, KALLIONIEMI A, KURISU W, THOR A, CHEN LC, SMITH HS, WALDMAN FM, PINKEL D AND GRAY JW. (1992). ERBB2 amplification in breast cancer analyzed by fluorescence in situ hybridization. *Proc. Natl Acad. Sci. USA*, **89**, 5321-5325.

KIBBELAAR RE, KOK F, DREEF EJ, KLEIVERDA JK, CORNELISSE CJ, RAAP AK AND KLUIN PM. (1993). Statistical methods in interphase cytogenetics: an experimental approach. *Cytometry*, **14**, 716-724.

KIRCHWEGER R, ZEILLINGER R, SCHNEEBERGER C, SPEISER P, LOUASON G AND THEILLET C. (1994). Patterns of allele losses suggest the existence of five distinct regions of LOH on chromosome 17 in breast cancer. *Int. J. Cancer*, **56**, 193-199.

KOTLIAR S, CAJULIS R, HAINES K, O'GORMAN M AND HIDVEGI D. (1993). Comparative study between flow cytometric analysis of DNA content and detection of numerical chromosomal abnormalities by fluorescence in situ hybridization of interphase nuclei with chromosome specific probes on fine needle aspirates of breast carcinomas (abstract). *Mod. Pathol.*, **6**, 31A.

LE BEAU MM. (1993). Detecting genetic changes in human tumor cells: have scientists 'gone fishing'? *Blood*, **81**, 1979-1983.

MATSUMURA K, KALLIONIEMI A, KALLIONIEMI O, CHEN L, SMITH HS, PINKEL D, GRAY J AND WALDMAN FM. (1992). Deletion of chromosome 17p loci in breast cancer cells detected by fluorescence in situ hybridization. *Cancer Res.*, **52**, 3474-3477.

MENARD S, SQUICCIARINI P, LUINI A, SACCHINI V, ROVINI D, TAGLIABUE E, VERONESI P, SALVADORI B, VERONESI U AND COLNAGHI MI. (1994). Immunodetection of bone marrow micrometastases in breast carcinoma patients and its correlation with primary tumour prognostic features. *Br. J. Cancer*, **69**, 1126-1129.

MICALE MA, VISSCHER DW, GULINO SE AND WOLMAN SR. (1994). Chromosomal aneuploidy in proliferative breast disease. *Hum. Pathol.*, **25**, 29-35.

MITELMAN F. (ed.) (1991). *Guidelines for Cancer Genetics. An International System for Human Cytogenetic Nomenclature (ISCN)*. Karger: Basle.

PINKEL D, STRAUME T AND GRAY JW. (1986). Cytogenetic analysis using quantitative, high-sensitivity, fluorescence hybridization. *Proc. Natl Acad. Sci. USA*, **83**, 2934-2938.

PIRC-DANOEWINATA H, CHOTT A, ONDERKA E, DRACH J, SCHLÖGL E, JÄGER U, THALHAMMER F, NOWOTNY H, ARYEE D, STEGER GG, LOCKER G, MICHL I, DJAVANMARD M, SIMONITSCH I, RADASZKIEWICZ T, HANAK H, SCHNEIDER B, HEINZ R AND MAROSI C. (1994). Karyotype and prognosis in non-Hodgkin lymphoma. *Leukemia*, **8**, 1929-1939.

RODRIGUEZ DE CASTRO F, MOLERO T, ACOSTA O, JULIA-SERDA G, CAMINERO J, CABRERA P AND CARRILLO T. (1994). Value of DNA analysis in addition to cytological testing in the diagnosis of malignant pleural effusions. *Thorax*, **49**, 692-694.

SHAY JW, WRIGHT WE AND WERBIN H. (1993). Toward a molecular understanding of human breast cancer: a hypothesis. *Breast Cancer Res. Treat.*, **25**, 83-94.

TAKAHASHI S, QIAN J, BROWN JA, ALCARAZ A, BOSTWICK DG, LIEBER MM AND JENKINS RB. (1994). Potential markers of prostate cancer aggressiveness detected by fluorescence in situ hybridization in needle biopsies. *Cancer Res.*, **54**, 3574-3579.

TAKITA K, SATO T, MIYAGI M, WATATANI M, AKIYAMA F, SAKAMOTO G, KASUMI F, ABE R AND NAKAMURA Y. (1992). Correlation of loss of alleles on the short arms of chromosomes 11 and 17 with metastasis of primary breast cancer to lymph nodes. *Cancer Res.*, **52**, 3914-3917.

WINQVIST R, MANNERMAA A, ALAVAIKKO M, BLANCO G, TASKINEN PJ, KIVINIEMI H, NEWSHAM I AND CAVENEE W. (1993). Refinement of regional loss of heterozygosity for chromosome 11p15.5 in human breast tumors. *Cancer Res.*, **53**, 4486-4488.

WOLMAN SR. (1994). Fluorescence in situ hybridization: a new tool for the pathologist. *Hum. Pathol.*, **25**, 586-590.

ZAFRANI B, GERBAULT-SEUREAU M, MOSSERI V AND DUTRILLAUX B. (1992). Cytogenetic study of breast cancer: clinicopathologic significance of homogeneously staining regions in 84 patients. *Hum. Pathol.*, **23**, 542-547.